# Supplementary material for: CoGTEx: Unscaled system-level coexpression estimation from GTEx data forecast novel functional gene partners
Source: PLoS One. 2024 Oct 4;19(10):e0309961. doi: 10.1371/journal.pone.0309961 (PMC11451983; doi:10.1371/journal.pone.0309961)
Supplement: S1 File — (DOCX) [file pone.0309961.s001.docx]

Supplementary Information for “CoGTEx: Unscaled coexpression estimation from GTEx data forecast novel functional gene partners”

Miguel-Angel Cortes-Guzman^1^ and Víctor Treviño^1,2,*^

^1^ Tecnologico de Monterrey, Escuela de Medicina, Bioinformática. Monterrey, Nuevo León, 64710 México.

^2^ Tecnologico de Monterrey, The Institute for Obesity Research. Monterrey, Nuevo León, 64849 México.

* Corresponding author: vtrevino@tec.mx


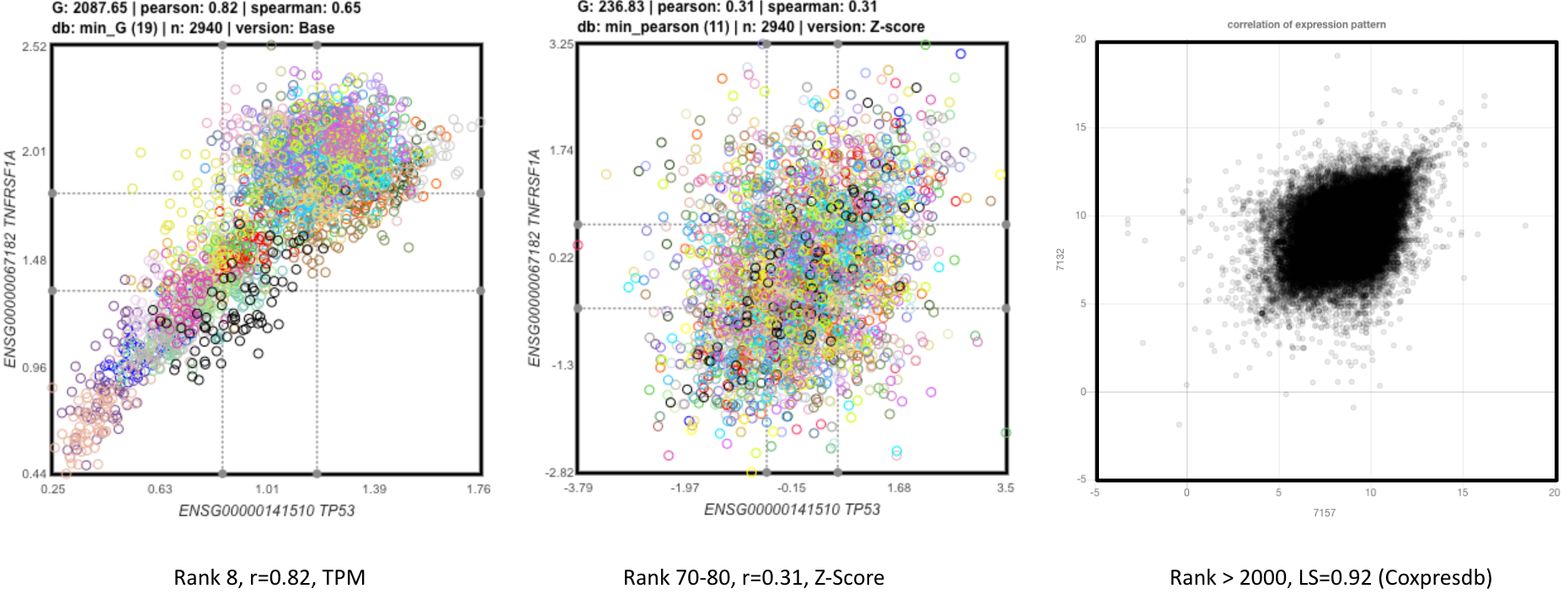


**Supplementary Figure 1. An example of overlap among top 100 coexpressed genes in both modalities**. The gene TNFRSF1A (vertical) is coexpressed with TP53 (horizontal) at the system-level (left, rank 8 in CoGTEx) and at the tissue level (center, rank ~ 70 in CoGTEx). COXPRESdb is also shown for comparison (right).

**
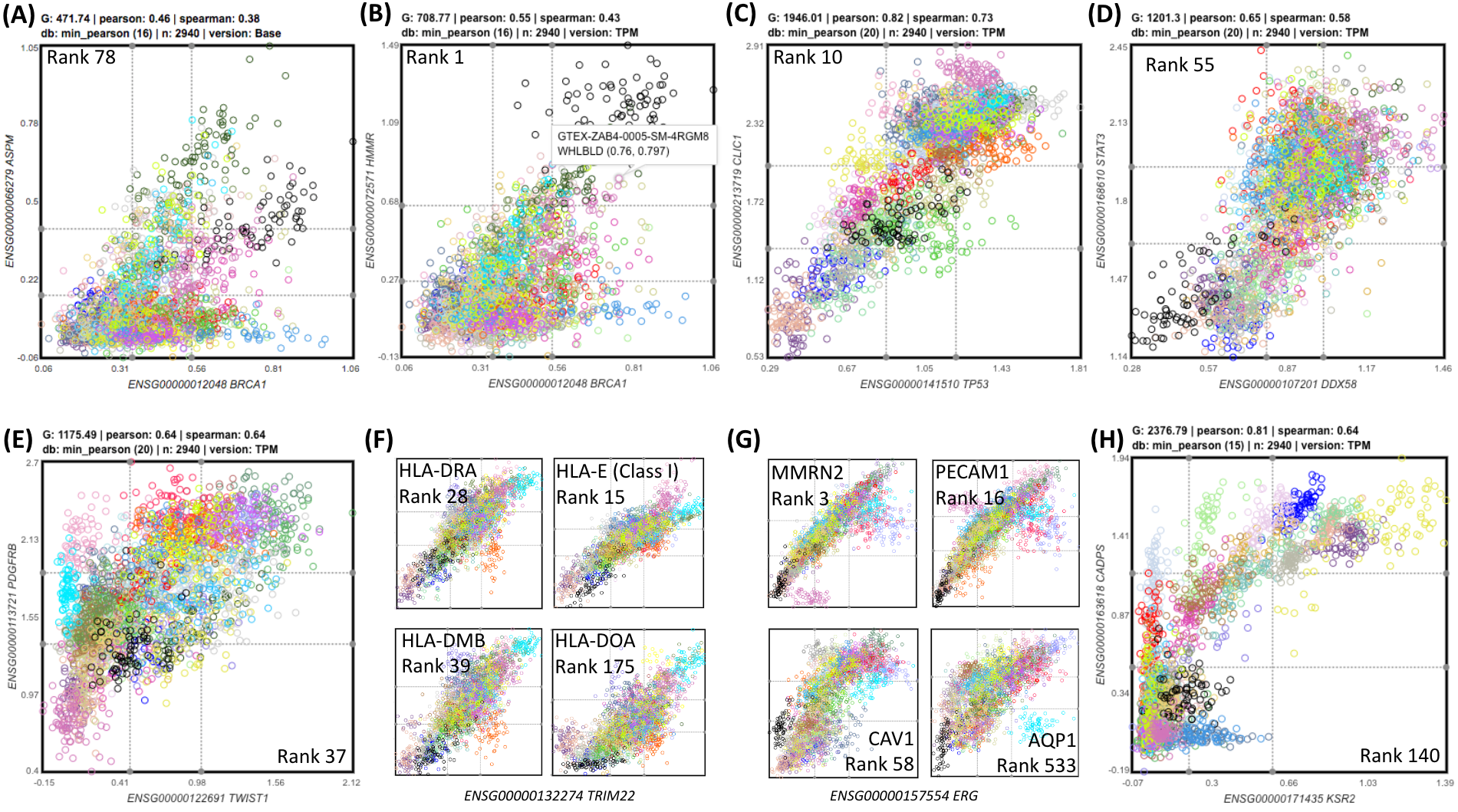
**

**Supplementary Figure 2. Examples of system level coexpression supported by literature**. (A) BRCA1-ASPM. (B) BRCA1-HMMR. (C) TP53-CLIC1. (D) DDX58-STAT3. (E) TWIST1-PDGFRB. (F) TRIM22-(various HLAs). (G) ERG-(various). (H) KSR-CADPS.


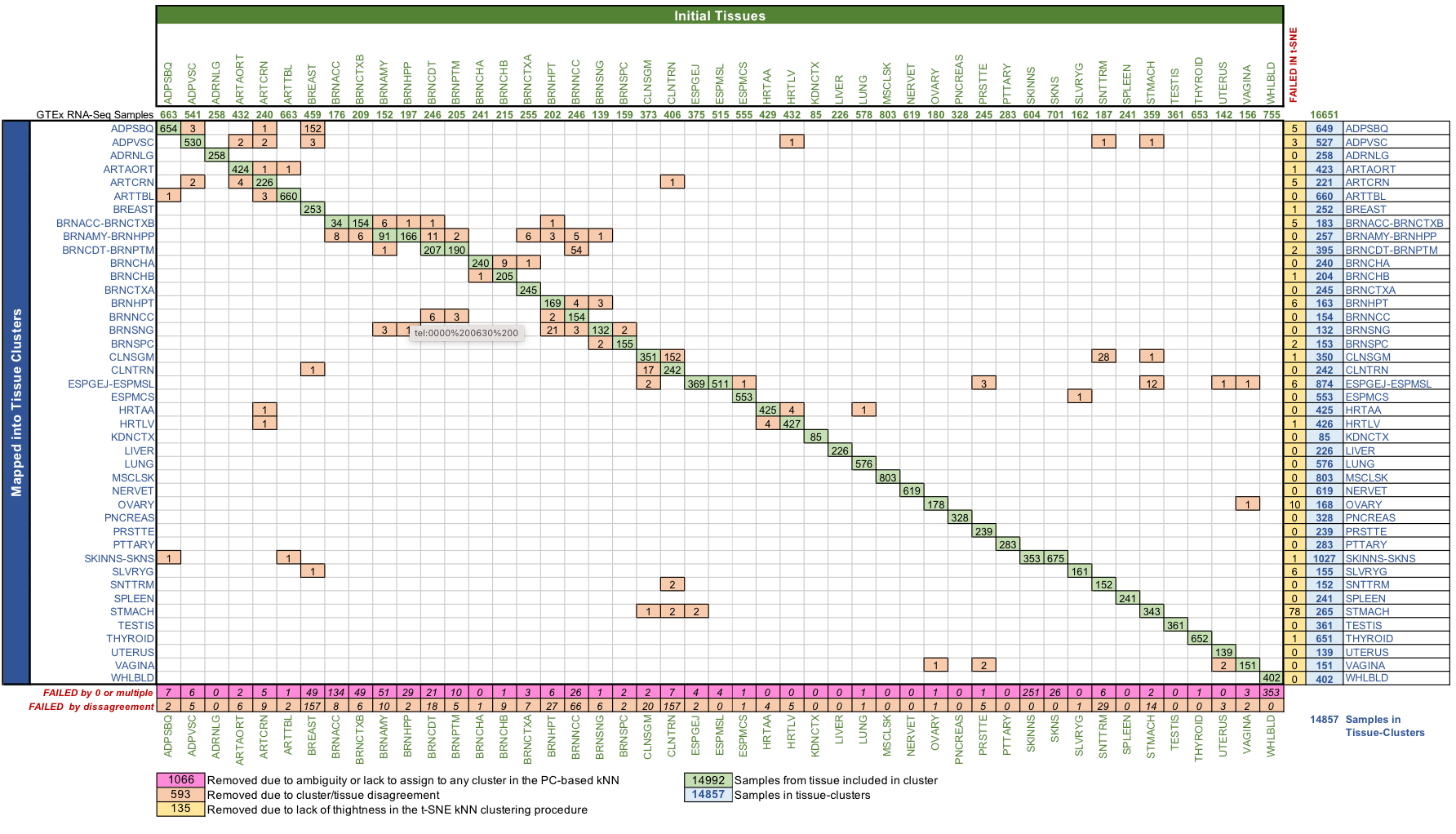


**Supplementary Figure 3. Generation of tissue clusters by sample filtering and curation.** GTEx tissues used are shown in columns. Original samples are shown above in green. Three procedures were employed to purify tissues. First, by removing those whose PC data disagree relative to its tissue (orange). Second, by removing those not assigned to a specific tissue (pink). By t-SNE coordinates, 10 tissues were highly mixed and therefore merged into 5 tissue clusters as shown on the right. The third filter removed those samples appearing far from their t-SNE cluster (yellow). The number in each cell corresponds to the number of samples designated in that cluster or procedure. The total number of samples per tissue-cluster is shown on the right. Starting from 16651 from 47 tissues (columns), we finally used 14857 samples assigned to 42 tissue-clusters (rows).


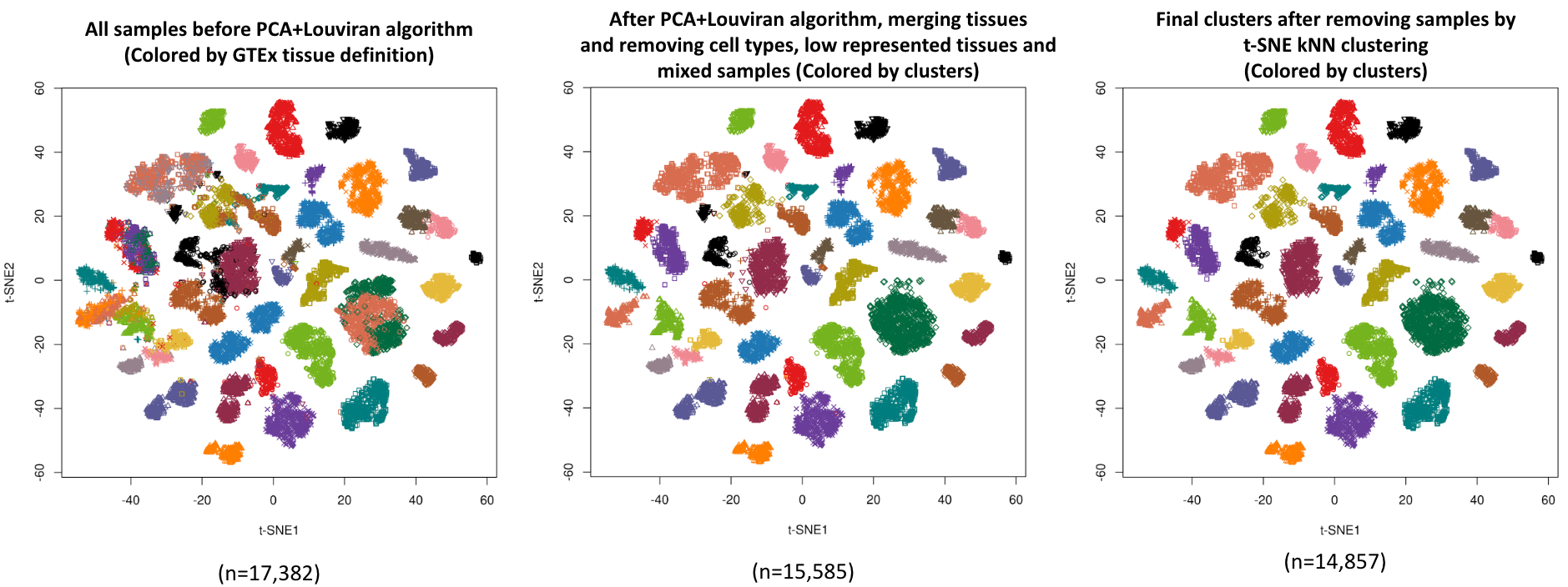


**Supplementary Figure 4. Filtering and curtains samples to generate tissue clusters as viewed in t-SNE coordinates.**


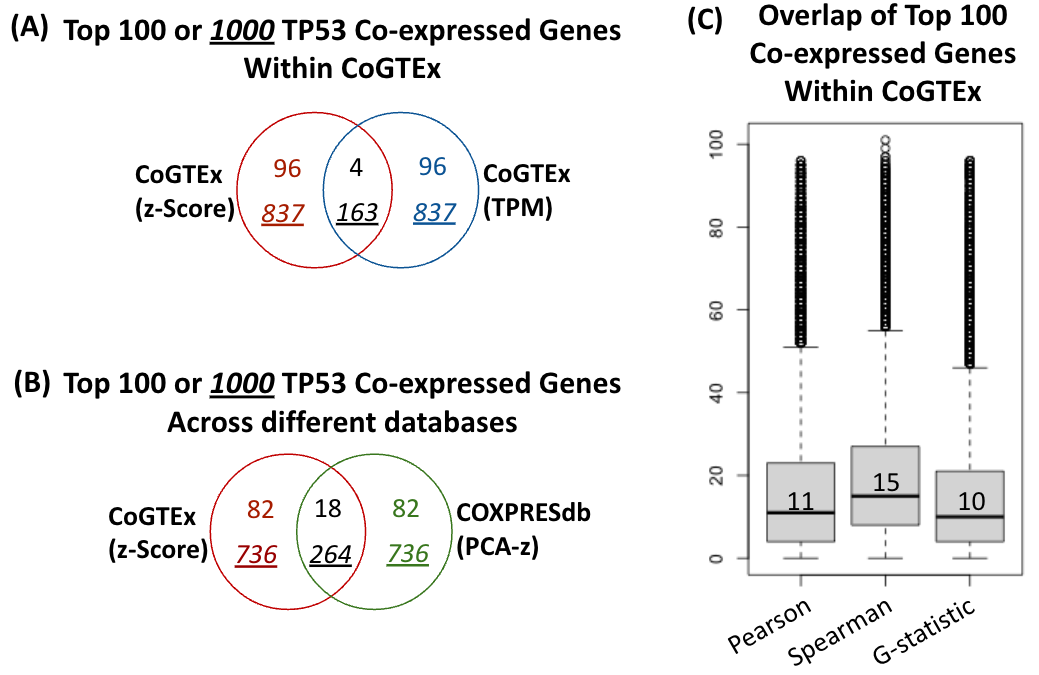


**Supplementary Figure 5. Overlap of “top” genes between system-level and tissue-level estimations.** (A) Example of top genes co-expressed with TP53 in CoGTEx between TPM and z-Score. (B) Example of top genes co-expressed for TP53 between CoGTEx (in z-score) and COXPRESdb. (C) Systematic comparison of all genes within CoGTEx between TPM and z-Scores.

**
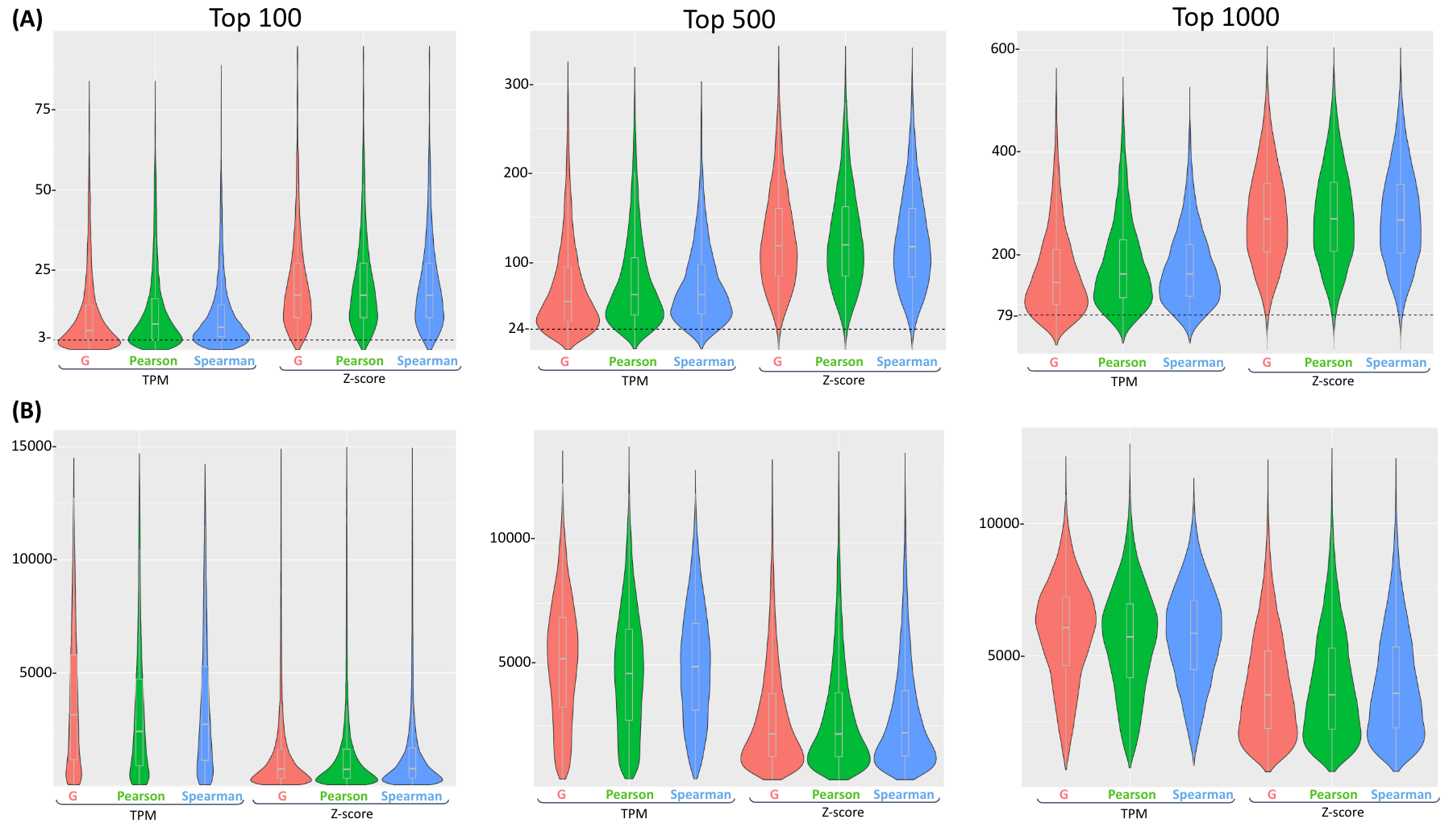
**

**Supplementary Figure 6. Comparisons of coexpression estimations at various top number of genes.** Panel A shows the distribution of the number of overlapped genes among the top 100, 500, or 1000 with those in COXPRESdb. Dashed lines correspond to significant association after FDR correction. Panel B shows the distribution of the median rank for the top 100, 500, or 1000 genes in COXPRESdb.

**Comparison of normalization methods**

We performed a comparison of normalization methods to show that our pre-processing does not generate large artifacts. For every tissue, we used the original raw (log10) data from 1000 random genes and the samples utilized after our pre-processing. Then we normalized the original raw-log10 data per tissue using Upper Quartile method (<https://doi.org/10.1371/journal.pone.0263344>) and Quantile Normalization. Finally, we added our data, after processing, for comparison. We then calculated the Pearson correlation matrix of these 1000 genes.

The figures included in below represent a comparison of the data (Top Panels) showing genes in rows and samples in columns. Rainbow colored lines at the left part of the heatmap represent the average expression (red=low, green=medium, blue to pink=high). Data were then normalized per tissue using Upper Quartile and Quantile Normalization. At right it shows our data after normalization and batch correction. The order of samples (columns) and genes (rows) are conserved as in the left panel.

Bottom panels shows the correlation matrix obtained from the 1,000 genes chosen (cyan to white to orange indicates correlation from negative (-1), zero, and positive(+1)). The rainbow-colored lines at top and left of each correlation matrix represent the average expression of that gene according to top-left panel. The correlation-matrix at the left is clustered by Euclidean distance and ward agglomeration. The 2^nd^, 3^rd^, and 4^th^ correlation-matrices conserve the ordering of the genes in the left-matrix for comparisons.

Overall, this comparison shows that:

(1) A normalization is indeed required since the data before processing contain many artifacts that deviate the correlation matrix toward positive values. (Plots at left)

(2) Upper Quartile Normalization per tissue (UQNT) provide a very similar results compared with Quantile-Normalization per tissue (QNT). Both resulting in softening the correlation matrix. (The second and third plots)

(3) The global Quantile-Normalization performed in our study still is quite similar to those results observed in UQNT and QNT. Moreover, our data shows less artifacts (expected due to the additional Batch Correction performed). For example, in ADRNLG, BRNSPC, HRTLV, MSCLSK, WHLBLD.
