# Supplementary material for: CoGTEx: Unscaled system-level coexpression estimation from GTEx data forecast novel functional gene partners
Source: PLoS One. 2024 Oct 4;19(10):e0309961. doi: 10.1371/journal.pone.0309961 (PMC11451983; doi:10.1371/journal.pone.0309961)
Supplement: Attachment — Submitted filename: Point-by-point answers CoGTEX PLOS-One-rev3.pdf [file pone.0309961.s003.pdf]

Point-by-point answers to reviewers' comments for the manuscript PONE-D-24-02309 entitled "CoGTEx: Unscaled system-level coexpression estimation from GTEx data forecast novel functional gene partners" received by email on July 16 2024.

In the following, the original reviewer comment is shown in black while authors answers are shown in blue and manuscript corrections in red.

Reviewer #1: The manuscript of Cortes-Guzman et al describes a large-scale gene coexpression analysis based on GTEx v8 gene expression data and the development of an online webtool for the exploration of results. The strong points of this work are the in-depth quality control and preprocessing of the GTEx data, as well as the calculation of global and tissue-level correlations using multiple metrics and association with other traits.

The manuscript is overall well-written with a sound methodology, however I have some comments regarding the authors selection of normalisation method and lack of comparisons with more coexpression webtools, apart from COXPRESdb. Please, find my comments in detail below:

Major Comments

i) The authors performed an elaborate pipeline for both gene and sample filtering. Indeed, quality-controlled samples result in higher quality coexpression networks. However, in this case, it is difficult to gage how much the preprocessing procedure aided towards that goal, as the expression data were initially normalised using Quantile Normalisation. The authors themselves present Johnson et al. [48] conclusions as follows: "between-sample normalization has the highest impact on building coexpression networks. Specifically, they showed that quantile normalization might have detrimental effects, perhaps due to extreme value adjustments.". Additionally, Hicks et al. (https://doi.org/10.1186/s13059-015-0679-0) mention that applying global normalisation methods (such as quantile normalisation) in samples of different tissues has the potential to remove biologically driven variation, leading to increased bias and mean squared error in downstream analyses. Moreover, Vandenbon (https://doi.org/10.1371/journal.pone.0263344) showed that Quantile Normalisation preprocessing resulted in the lowest-quality coexpression networks overall. Thus, the authors should consider another normalisation method, perhaps a per-tissue method, such as smooth quantile normalisation or tissue-aware

normalisation (https://doi.org/10.1186/s12859-017-1847-x) also tested on GTEx data, and whether that could improve the correlations and, as such, the performance of their tool.

>>>>Authors Response:

We agree with reviewer that the normalization procedure is an important aspect of our study. We also agree with reviewer (and authors of the referred papers) that the closer to the quantile normalization assumptions the better.

To show that our normalization procedure is, we performed two verification procedures.

First, we compared the densities per tissue of the original data of the samples used after tissue-cluster generation. Overall, the densities are quite similar, perhaps except for Testis.

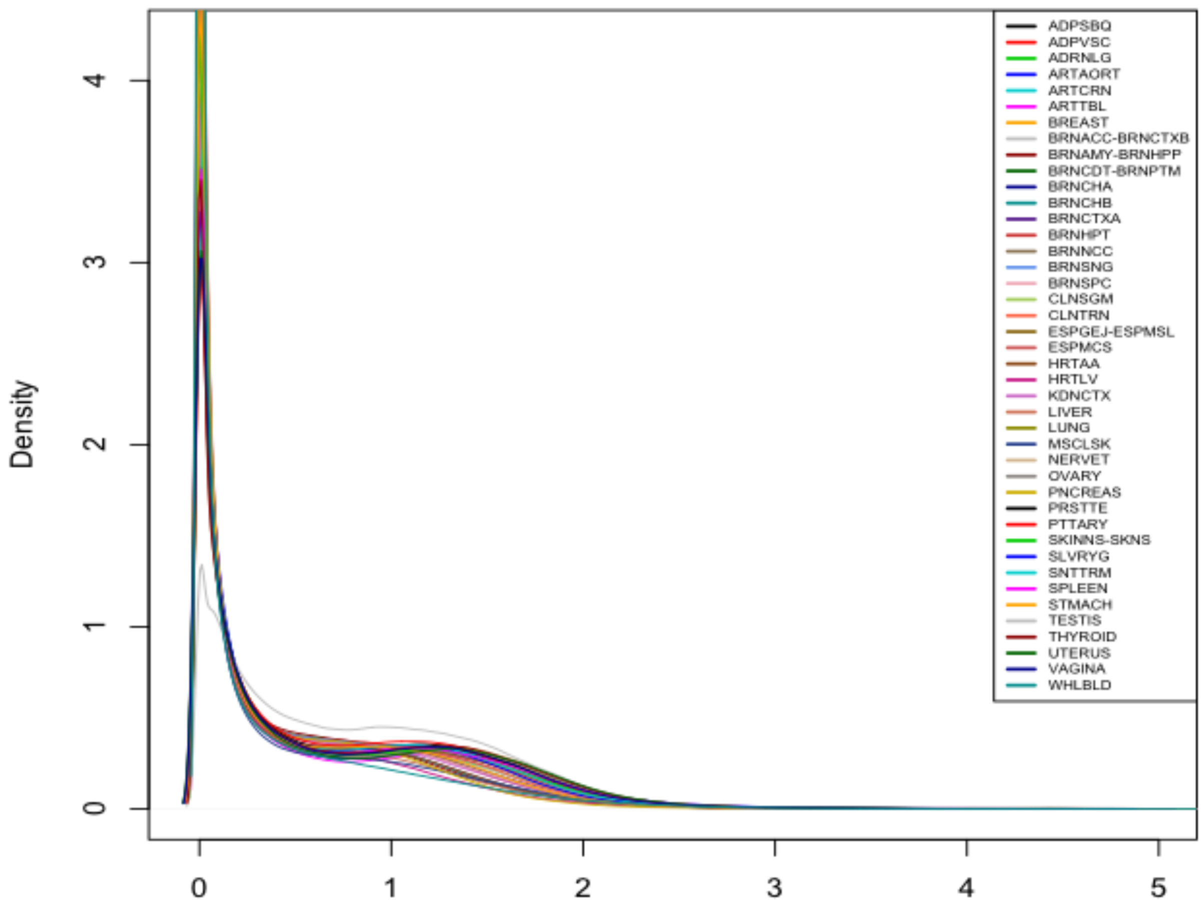

Response Figure 1. Density of raw-log10 transformed data from each tissue before batch correction and normalization.

Second, we compared the effect of normalization on the correlation matrix. We used the final samples from each tissue and the original raw RNA-Seq count data scaled by Log10+1

as usual and tested, Upper Quartile Normalization per tissue, Quantile Normalization per tissue, and our final data used in this study after global quantile normalization and batch correction. We used Upper Quartile Normalization for comparison because it was the best normalizing procedure obtained by Hicks *et al*. We compared the data of the top 1000 genes and the Pearson correlation matrix derived from the data.

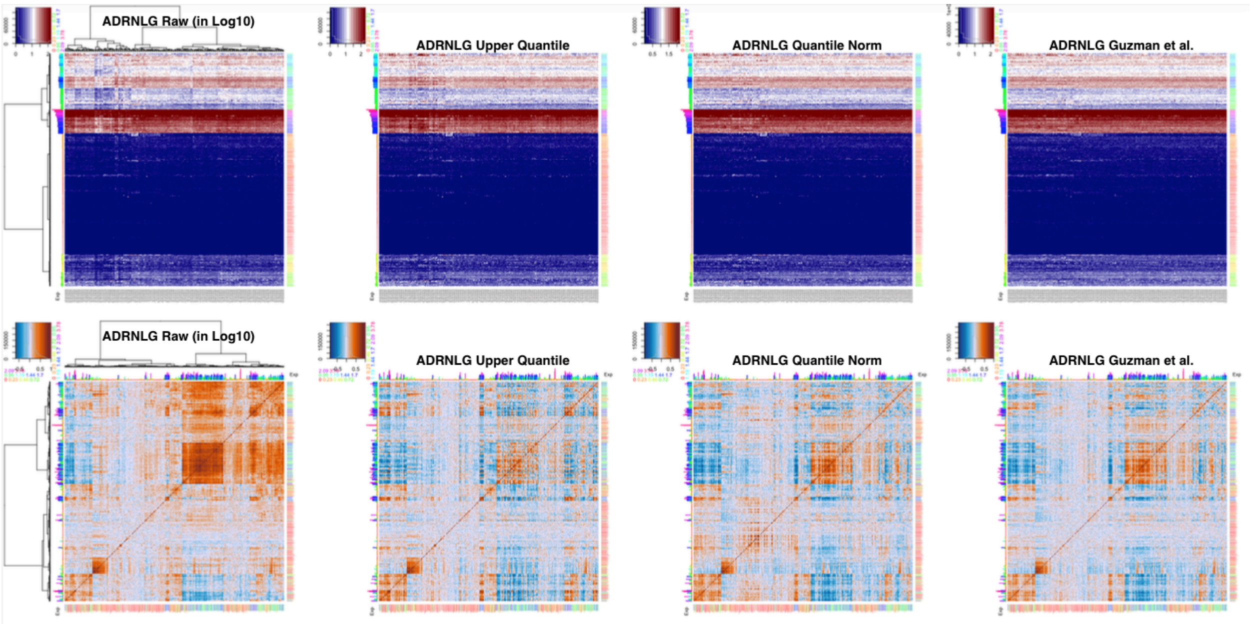

Response Figure 2. Example of the effect of normalization. Top panels. Left show raw (log10) data from 1000 random genes in rows and samples in columns. Rainbow colored lines at the left part of the heatmap represent the average expression (red=low, green=medium, blue to pink=high). Data were then normalized per tissue using Upper Quartile and Quantile Normalization. At right it shows our data after normalization and batch correction. The order of samples (columns) and genes (rows) are conserved as in the left panel. Note that artifacts are successfully corrected in all methods. Bottom panels shows the correlation matrix obtained from the 1,000 genes chosen (cyan to white to orange indicates correlation from negative (-1), zero, and positive(+1)). Note that the artifacts in unnormalized expression (top,left) generates higher artificial correlations that are removed in all normalization methods.

Overall, the results show that :

(1) A normalization is indeed required since the data before processing contain many artifacts that deviate the correlation matrix toward positive values. (Plots at left)

(2) Upper Quartile Normalization per tissue (UQNT) provide a very similar results compared with Quantile-Normalization per tissue (QNT). Both resulting in softening the correlation matrix. (The second and third plots)
(3) The global Quantile-Normalizaton performed in our study still is quite similar to those results observed in UQNT and QNT. Moreover, our data shows less artifacts (expected due to the additional Batch Correction performed). For example, in ADRNLG, BRNSPC, HRTLV, MSCLSK, WHLBLD.

Still we recognize this issue worth more discussion and added in discussion:

> "While this manuscript was in preparation, the study by Johnson *et al.* was published [48]. They concluded that between-sample normalization has the highest impact on building coexpression networks. Specifically, they showed that quantile normalization might have detrimental effects, perhaps due to extreme value adjustments. Nevertheless, Johnson *et al.* tested tissue-specific networks, and we used a highly robust methods to estimate correlation that considers a subset of samples that may alleviate the extreme-value issue. For example, by looking at system-level coexpressions in Figure 5, it seems rather unlikely that the coexpression estimation will be different when some of the extreme values may be altered by using a different normalization scheme. Thus, it may be possible that the effect observed by Johnson *et al.* does not apply in general in our system-level setting. Moreover, we compared raw data, upper quartile normalization per tissue (as proposed by [49]), quantile-normalization per tissue, and our data after processing (see Supplementary Material) showing that our processing shows comparable results to those normalizations performed at the tissue level. Still, we plan to consider in future versions the Johnson *et al.* recommendations and those from other authors [49–51] focusing on differences compared to the calculations presented here. Based on our robust estimation and that correlation does not seem to be altered by extreme values, we anticipate that differences with current estimations will be minor in the system-level calculations.".

We added the comparison of all tissues in the supplementary material. We appreciate this comment that improved the manuscript and provided insights for future versions of our estimations.

ii) The authors performed a comparison of their correlations with those of COXPRESdb, another popular coexpression database, checking the overlaps of the top 100 partners for each gene, between COXPRESdb and CoGTEx, and showing only a 15% average gene overlap. Is it possible for the authors to comment whether their method ultimately outperforms the one used by COXPRESdb?

>>>>Authors Response:

We cannot declare that we outperform other calculations, in particular those of CoXPRESdb. Although it can be tested whether different estimations of coexpression are “more precise”, all these comparisons are based on a “known” reference, that is, the cummulative knowledge of interactions or regulations. Nevertheless, this is far from perfect precison and accuracy and are highly biased to the how the knowledge is being accumulated. Put it plainly, we don’t have the whole ground truth of gene coexpression. Indeed, our results confirm that z-scores per tissue are more accurate than TPM estimations, so, basically CoexpresDB should ‘outperform’ CoGTEx in terms of ‘accuracy’ compared to current knowledge. Nevertheless, this only shows that our knowledge is based on “relative” experimental assays within a few cell-lines or tissues (similar to tissue-based estimations or z-scores) instead of what is going on in an organism. With the every-day coming massive experiments and efforts (such as those from GTEx), novel approaches are needed to view the data in different ways. We show that our estimation provides a different view, that of observing an organism as a whole and the aspects of gene expression along all tissues in the organism not only in a particular tissue. We show that estimations makes sense and we hope that our view and accompaining tool will help to discover more biological knowledge.

In summary, the content and estimations of coexpression are different between CoGTEX and CoexpresDB. Our focus is to show that the estimation provided by CoGTEX are (1) different and (2) useful.

Furthermore, the authors mention several GTEx-based coexpression works in the introduction, claiming that "researchers have no resources to view and analyze GTEx gene coexpression partners easily". However, webtools, such as GeneFriends (mentioned in the manuscript) and HGCA (not mentioned in the manuscript, https://doi.org/10.3390/cells12030388), offer results from large-scale gene coexpression analyses based on the same GTEx data, while also offering visualisation options (Networks or Dendrograms respectively), and seamless enrichment analyses. Since the authors already used COXPRESdb in benchmarking, it would be interesting to see how CoGTEx fares compared to those two other GTEx-based webtools.

>>>>Authors Response:

Today (19/Jul/2024), despite GeneFriends interfase is quite interesting, it does not provide a comparison of the raw data. For example using POMC & TBX19 genes and GTEX data in GeneFriends provide “a network” connecting both genes by 0.8821 of correlation, however the data and/or procedures that generated the result (r=0.8821) cannot be seen. Our estimation in TPM for POMC and TBX19 is 0.42 and in Z-score is 0. Both of which are

different to GeneFriends. In CoGTEX, the data can be shown as a scatter plot in the website and the data can be even downloaded. Similarly in HGCA (https://www.michalopoulos.net/hgca2.0/), which it show a dendrogram of local coexpression but it does not show the actual calculations of coexpression between genes. So, although the "partners" can be viewed, the analysis of the data is not provided. Thus the user have to "believe" what it is provided. We think that looking the actual data makes a lot of difference to judge whether the information "makes sense".

Therefore, to clarify the sentence regarding "partners", we updated the phrase and added reference to HGCA:

> "Despite some efforts [25–29], researchers have no resources to view and analyze the data of GTEx gene coexpression estimations easily.".

iii) For their Enrichment benchmarking, the authors could also consider the three aspects of Gene Ontology, apart from KEGG pathways and GIANT networks. Furthermore, in the results section "Estimated system-level coexpressions can be validated", the authors could enhance their examples by adding external enrichment analysis of multiple coexpressed partners (adding enriched GO terms or Pathways), rather than just focusing on one-pair relationships. As the main aim of coexpression webtools is to replicate known biology, the best way to ascertain the validity of the coexpressed gene partners to a single gene, it to see whether the enriched biological terms of the coexpressed genes are related to the bibliography-supported input gene's biological functions. In any case, we expect the authors to comment on the relevance of the enriched KEGG terms of the top coexpressed genes and the selected gene.

>>>>Authors Response:

For the enrichment benchmarking, the idea of using KEGG Pathways and GIANT networks is to show that results make sense and can be useful. Thus, adding GO to the comparison would not add so much.

For the use of GO enrichments for validation in addition to gene-pairs, we appreciate the comment and added an analysis of BRCA1 coexpressed genes as an example of validation of biological functions. As a result, we also added a Table. The new paragraph is:

> "Other common use of coexpressed genes is to inform gene functions, biological processes, or pathways in which a gene may be involved. To illustrate this, we performed a gene set enrichment analysis using EnrichR [48] from the top 100 coexpressed genes of BRCA1. Particularly, we used the gene ontology (GO) terms from the top 10 Biological Processes (BP) derived from the top 100 coexpressed genes according to the tissue-level estimation

compared to the top 10 BP from those coexpressed genes estimated by system-level. Note that a certain degree of overlap in the results is expected since 24 genes are present in both lists of top 100 genes, perhaps, at different level of importance. Table 2 shows the results of the top 10 BP resulted in each analysis. Interestingly, using the top genes from the tissue-level estimation, the top BP tended to be related to the important role of BRCA1 in DNA repair of double strand breaks while the top system-level coexpressed genes highlight the role of BRCA1 in spindle-kinetochore-microtubule structures [49,50]. Although many of these BP can be obtained from either, tissue- or system-level rank of genes, this analysis validates the functional information and highlight different aspects of known biology, which can be used for discovery too.".

iv) Could the authors elaborate more on how they computed the z-score for the tissue-level modalities in the methods section "Standardized database version"?

>>>>Authors Response:

We amended the referred section to clarify the computations. The new paragraph is:

"**Standardized database version.** Additional to the main CoGTEx database, which is calculated from TPM-scale gene expression values, we have also repeated calculations using standardized data by tissue cluster for comparative purposes with other databases such as COXPRESdb. This is referred as Z-Scores in our web application. For this, TPM gene expression in log10 scale was transformed to z-scores by $z = (x - m) / s$, where m is the mean expression of the gene in a particular tissue, and s is the sample standard deviation of the gene in the tissue, and x represent the gene expression of a sample in the tissue.".

v) The authors should clarify in the text when the signed and when the absolute correlations are used. For example, in the method section "Enrichment of biological terms supports coexpression estimations", it is not clear if the top and bottom coexpressed partners are determined using the absolute correlation values or if the anti-correlated genes (negative correlations) are considered as the least coexpressed ones. Same applies to the web platform itself.

>>>>Authors Response:

We appreciate that the reviewer spotted this omission. We amended the paragraph as:

"Thus, for any gene, we calculated the pathway associations between its top 5% most coexpressed genes (1672 genes, absolute coexpression) compared to the bottom 5% less coexpressed gene (those genes closer to zero coexpression).".

We also amended the legend of the Figure 3 to clarify this.

Web platform

i) The web interface is simple and fast, however, I was not able to get the Coexpressed

genes rank comparison plot to work.

>>>>Authors Response:

We appreciate that the reviewer spotted this omission. We amended the paragraph as:

"We added a tool to analyze the ranks of computed metrics to identify these exceptions (option in Analyze tab within the gene page). "

"We added a tool to analyze the ranks of computed metrics to identify differences between system- and tissue-level coexpression described in this manuscript (option in Analyze tab within the gene page). "

ii) In addition, it would be nice if the authors could add a link or button to automatically submit the list of coexpressed genes to other websites for enrichment analysis, such as StringDB or EnrichR.

>>>>Authors Response:

We amended the section "CoGTEx database and web accessibility" including:

"The coexpressed gene list web page is searchable with associations for all metrics. Associations based on covariate levels are also included in this table (see Methods). The listed tables can be easily copied or exported, which facilitate further user analyses such as gene enrichment or network reconstructions.".

iii) Finally, adding a tutorial or FAQ to guide less technologically adept users (that would include Biologists who would be the main users of the webtool) would also be nice.

>>>>Authors Response:

We prepared the webapp to include a proper FAQ/tutorial in the landing page. It is quite brief but we intend to grow this with time and depending on user feedback. A reference to the corresponding author e-mail is included to allow users to ask for help.

We appreciate this comment.

Minor Comments

i) The authors should update their citations for all webtools and databases mentioned, to match the latest corresponding publications. One such example is COXPRESdb, with the most recent publication being the following: https://doi.org/10.1093/nar/gkac983

>>>>Authors Response:

As recommended, we added the newest reference for coxpresdb.

ii) The authors should perform a more thorough proof-reading of the manuscript as there are certain typos, e.g. CoGTEx is spelled as CoGTex, CoGTeX and CoGTEX in multiple cases.

>>>>Authors Response:

As recommended, we revised and amended all references to CoGTEx.

We appreciate all coments from reviewer 1 that helped to improve our manuscript.
