# Supplementary material for: CoGTEx: Unscaled system-level coexpression estimation from GTEx data forecast novel functional gene partners
Source: PLoS One. 2024 Oct 4;19(10):e0309961. doi: 10.1371/journal.pone.0309961 (PMC11451983; doi:10.1371/journal.pone.0309961)

Point-By-Point answers to reviewers' comments for the manuscript **PONE-D-24-02309R1** entitled "CoGTEx: Unscaled system-level coexpression estimation from GTEx data forecast novel functional gene partners" received by email on Aug 6th 20224.

In the following, the original reviewer comment is shown in black while authors answers are shown in blue and manuscript corrections in red.

Reviewer #1: I have read the revised version of Cortes-Guzman et al. The authors additionally performed an extensive analysis showing how their normalisation method fares with other normalisation methods shown to perform well for coexpression analysis. In addition, they added a global and local GO term analysis for BRCA1 coexpressed genes of CoGTEx.
However, in their response about the benchmarking with other similar webtools (GeneFriends and HGCA), they only studied one gene pair (TBX19 and POMC). My major comment lies with this and the subsequent comparison with other webtools that I originally asked for.
1) First of all, the authors only mentioned the interaction between TBX19 and POMC in the "response to reviewers" but they did not include this information in the text. The authors did not give any explanation as to why they selected that specific gene pair (TBX19 and POMC). Indeed, TBX19 is a tissue-specific regulator for POMC (PMID: 11447259).

>>>>Author's Response:

As the reviewer showed, indeed it is true that TBX19 is a regulator of POMC (as demonstrated by ~60 pubmed records of the query "TBX19 POMC" and many reviewed in reference https://pubmed.ncbi.nlm.nih.gov/26792828/), mainly in Pituitary and perhaps also in Hypothalamus but details in others tissues are not so clear. As expected, some tools may or may not detect this association.

Our response providing an example of the possible "coexpression" of POMC and TBX19 attempted to justify why we claimed that the data of GTEx coexpression estimations from others tools cannot be visualized. Without the visualization, one relies solely on the calculation given by the tool. With the visualization, the data can speak by itself.

We used the TBX19-POMC example NOT MENTIONED in the main text because those in the main text are already comprehensively analysed. We selected this gene-pair example because we are working in the lab with them, which is part of a separate research almost unrelated to this manuscript. We do not want to mention in the current manuscript

because (a) the coexpression of this gene pair is more important when it is analyzed by tissues than by system-level coexpression, (b) we are still working on it and there are unclear contexts, and (c) can be confusing for the readers.

Nevertheless, this gene-pair illustrates the point we wanted to make for the reviewer: "Looking at the data justify why one gene-pair can be interesting or not". In our case, the data that generates the correlation can be seen. We did not claim that POMC-TBX19 coexpression was among top in system-level, instead we claimed that "data can be viewed and downloaded".

Instead of POMC-TBX19, we added 3 gene analyses deriving in a new figure 7.

The coexpression of TBX19 and POMC is loosely supported in StringDB (https://string-db.org/interaction/9606.ENSP00000356795/9606.ENSP00000384092?c1=65ffa3&c2=ff0000), as putative homologs were coexpressed in other organisms. The authors reported that TBX19 and POMC had a 0.42 Pearson correlation in CoGTEx and ~0.88 in GeneFriends, but they failed to realize that HGCA did not discover this gene correlation as significant, as they expected the actual calculations of coexpression between genes.

>>>>Author's Response:

As the reviewer also noted there are different estimations among tools. The fact that the relation is loosely supported in String or not present in HCGA or any other tool does not mean that it is not relevant. We were not claiming relevance in the TBX19-POMC coexpression, instead we were referring to viewing the corresponding data, which can be useful when different tools provide, or not, a relationship.

The important point (for us) in the response was that estimations from diverse databases or tools are different and therefore viewing the data is important, which can be done in CoGTEX.

Ultimately, the things that matter to the end users (Molecular Biologists) when they use a coexpression tool to identify novel functional partners to a gene of interest, are the top ranked genes of a coexpressed gene list and the correlation cutoff, not the data and procedures that resulted in that list.

>>>>Author's Response:

We agree with reviewer that the most important aspect that matters to end users is the rank of top genes. Indeed, we emphasize this along the manuscript comparing the ranks from CoGTEx and other tools and we added another analysis comparing tools as shown in the new Figure 7.

Nevertheless, we differ with the reviewer in that end users do not appreciate data and/or procedures. We consider very important to show the scatter plots because we are providing a different view of coexpression (system-level). In that context, the scatter plot is a strong source of support. Without that, our provided calculation is "blind" to end users and could be misleading or can be seen as a weak support. Similar tools such as StringDB also provides end users on the details of the evidence that support a score (for instance the button "Show" in the POMC-TBX19 page sent by the reviewer which shows all textmining data supporint the score). Thus, some evidence may be appreciated by end users, which can be viewed in CoGTEX.

In CoGTEx, when the coexpressed genes for TBX19 are downloaded, POMC is ranked in 1020th position, while in the corresponding list for POMC, TBX19 is ranked as 185th. In their "response to reviewers", the authors do not study the top coexpressed partners of each of the two genes and whether these are bibliographically-supported functional partners, thus not stating if they consider that the 0.42 correlation of CoGTEx to TBX19 and POMC is something that should be expected or not.

>>>>Author's Response:

We already clarified that we did not claim that POMC-TBX19 coexpression was significant or among top in system-level, instead we claimed that "data can be viewed and downloaded" and this is the main reason we did not add this gene-pair in the main text.

To infer whether a webtool has the potential to identify new functional relationships, we can check the relationships between the gene of interest and its coexpressed genes one-by-one. The authors have already performed this, through a PubMed literature search approach. However, this method is not easily verifiable and it cannot be massively performed for the benchmarking of different webtools.

Instead, to facilitate the process of confirming whether the tool can replicate known biology, we can check if the enriched biological terms in the list of top coexpressed genes are related to the gene of interest's functions. This is easy to perform for multiple webtools. The authors have already used that approach on the top 100 local and global coexpressed partners of BRCA1, only for their own tool. In my previous comments, when referring to

benchmarking, I was expecting the authors to do the same thing, for the other similar webtools, and not just mention one single gene pair (TBX19 and POMC).

Taking all this into account, the authors should perform this benchmarking with CoGTEx and the other webtools (CoXPRESdb, GeneFriends and HGCA) by taking the top 100 or so coexpressed partners in each webtool, for 3-4 cases of genes with well-documented roles in bibliography. The authors can use EnrichR or any other enrichment webtool (StringDB, gProfiler, WebGestalt). The authors can refer to the paper of HGCA (PMID: 36766730), where that kind of benchmarking was performed (See Section 2.6. STRING Analysis and Section 3.2. Coexpression Tool Benchmarking).

>>>>Author's Response:

We already demonstrated that our estimation is biologically meaningful along the text. This can be shown in Figure 3, Figure 4, Figure 7, Figure 8, Table 1, Table 2 and their corresponding text in the manuscript.

Nevertheless,as suggested by the reviewer, we added the analysis of 3 genes comparing the enrichment analysis of CoGTEx, Coxpresdb, GeneFriends, and HGCA 2.0.

The analysis added value to the manuscript and clarify the utility of our approach.

We appreciate the reviewer suggestion.

For this point, we added a result paragraph and a new figure.

In results we added:

> "To provide a more comprehensive comparison, we performed the above analysis for C2orf68, MT1M, and NR3C1 genes that were also used for analysis and benchmarking in HGCA 2.0 [29]. In this comparison, we also added the top 100 genes provided by GeneFriends, CoxpresDB, and HGCA 2.0. We merged the top 10 GO BP terms per tool filtering the terms that include at least 3 genes. The results are shown in Figure 7. For C2orf68, our gene rank also associated top terms found by the other three tools related with Skin, Keratinocyte, and Epidermis. There were no exclusive terms found by system-level coexpression, thus, the top biological terms are consistent with the other tools. For MT1M, the EnrichR analysis of positive system-level coexpression rank also associated the top terms found by the other tools related to Zinc, Copper, and other metal ions. There were two exclusive top terms derived from our estimations, but these were observed at the tissue-level coexpression (CoGTEx Z in Figure 7), which were related to Lens Fiber differentiation. Metallothioneins, as MT1M, have been suggested to play a role in the eye [52][53], perhaps as antioxidant functions [54]. So, the rank from system-level coexpression does not generate GO BP terms (among the top 10) that were not supported by other tools, while the tissue-level provided a sensible prediction. For NRC31, all tools yielded exclusive GO BP terms. HCGA 2.0 showed exclusive terms for Autophagosome and Wnt Signalling, GeneFriends

for Mitotic Sister Chromatid and mRNA Splicing, CoxpresDB for DNA Replication, RNA Metabolism, and Protein Localization, and system-level coexpression (CoGTEx TPM in Figure 7) showed exclusive terms for Cardiac and Muscle Hypertrophy. A null mutant in zebrafish for the glucocorticoid receptor *nrc31* revealed heart abnormalities [55], suggesting that the association generated by the system-level coexpression could be useful. In summary, this analysis reveal that top system-level coexpressed genes provides sensible and useful gene set enrichment analyses.".

And the figure:

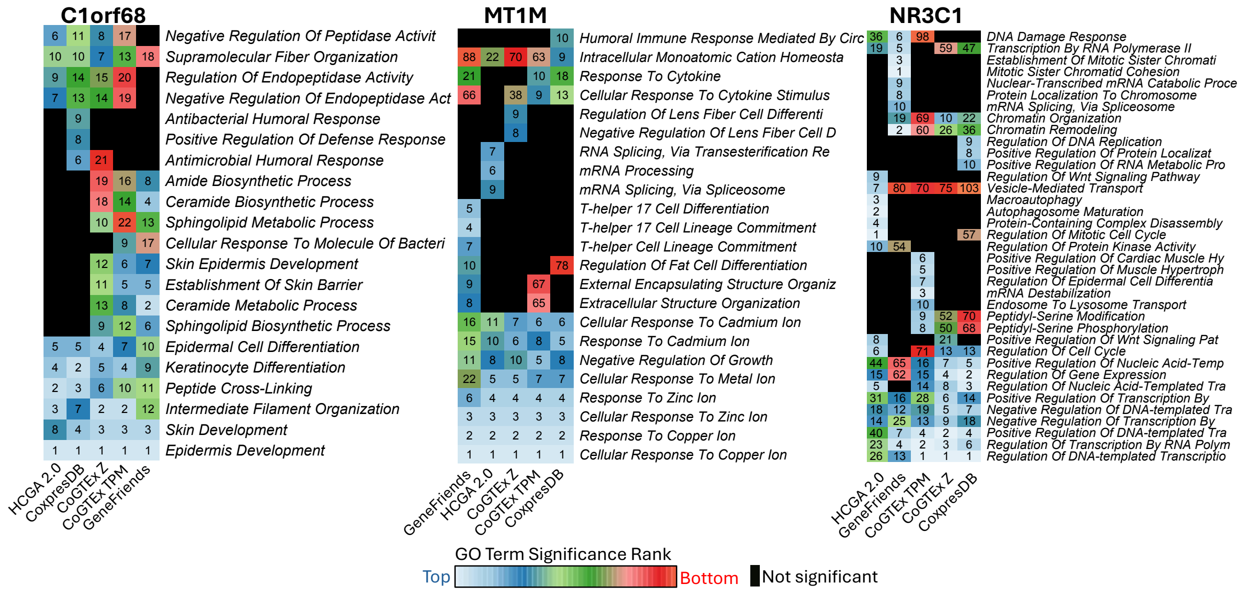

Figure 7. **Comparison of gene set enrichment analyses for three selected genes and four coexpression tools.** Each heatmap shows the rank of significant gene ontology terms for biological processes resulted by a EnrichR analysis from the top 100 coexpressed genes reported by the shown coexpression tools. Colors refer to the position of the rank for the corresponding biological term. Black refers to not significant GO terms or that did not meet the criteria.

2) In their "response to reviewers", the authors added a rationale for the difference in results with CoXPRESdb. They should add something like that in the discussion section of the main text, also including the other webtools they will use in the benchmark.

>>>>Author's Response:

As suggested. We added the paragraph (with amends) in discussion as:

"We cannot declare that system-level coexpression outperform other coexpression calculations, in particular those of z-score estimations as CoXPRESdb. Although it can be tested whether different estimations of coexpression are more precise than others, as we did with pathway databases, all these comparisons are based on a known reference. The reference is the cummulative knowledge of interactions or regulations and, in pathway cases, even knowledge that is selected for particular purposes. Nevertheless, this reference knowledge is far from perfect and is highly biased to how the knowledge is being accumulated. For example, many experiments are based on experimental assays within a few cell-lines or tissues (similar to tissue-based estimations or z-scores) instead of what happen in an organism as a whole. Thus, we do not have a comprehensive ground truth of gene coexpression to accurately evaluate tools. Indeed, our results confirm that z-scores per tissue seems more accurate than TPM estimations. So, basically CoXPRESdb (or per-tissue z-score estimations) should outperform system-level estimation in terms of accuracy compared to current knowledge. Nevertheless, this does not undermine our proposal. With the recent acquisitions of massive experiments and efforts (such as those from GTEx), novel approaches are needed to view the data in different ways. We show that our system-level estimation provides a different view, that of observing an organism as a whole and the aspects of gene expression along all tissues in the organism as opposite to observing a particular tissue or cell-line, or relative expression across tissues. We show that our estimations makes sense and we hope that our view and accompaining tool will help to discover more biological knowledge. In summary, the content and estimations of coexpression are different between CoGTEX and common tools for tissue-relative coexpression estimation from GTEx (such as CoXPRESdb [20,38], HGCA [29], GeneFriends [28]) and other tools such as StringDB [63]. Our focus is to show that the estimation provided by CoGTEX are different and useful."

We appreciate this comment that helped to make our discussion more interesting.

Minor Comment:

1) It is still not possible to run the "coexpressed gene rank comparison" plot in CoGTEx. It seems to load indefinitely when I choose "coexpressed gene rank comparison" option in the plot type and click the green cross icon next to the ENSG of a gene. I did not ask for the authors to amend the text, but to solve this issue in their website. Also, I am unable to find within the website what the authors mention as "option in Analyze tab within the gene page", unless they refer to the green cross icon next to each gene ENSG code.

>>>>Author's Response:

We apologize for the confusions. With the current description "coexpressed gene rank comparison" we realize the reviewer was referring to the main web page (instead of the per-gene page). Thus we revise it and indeed the page had a typo in one function of the javascript that caused the "load indefinitely" problem. This issue has now been corrected and the figure can be now visualized. As an example, we show below an example of the plot

for BRCA1, it takes around ~7 seconds to load (this figure is not added to the manuscript, it is shown here to demonstrate that the problem has been corrected).

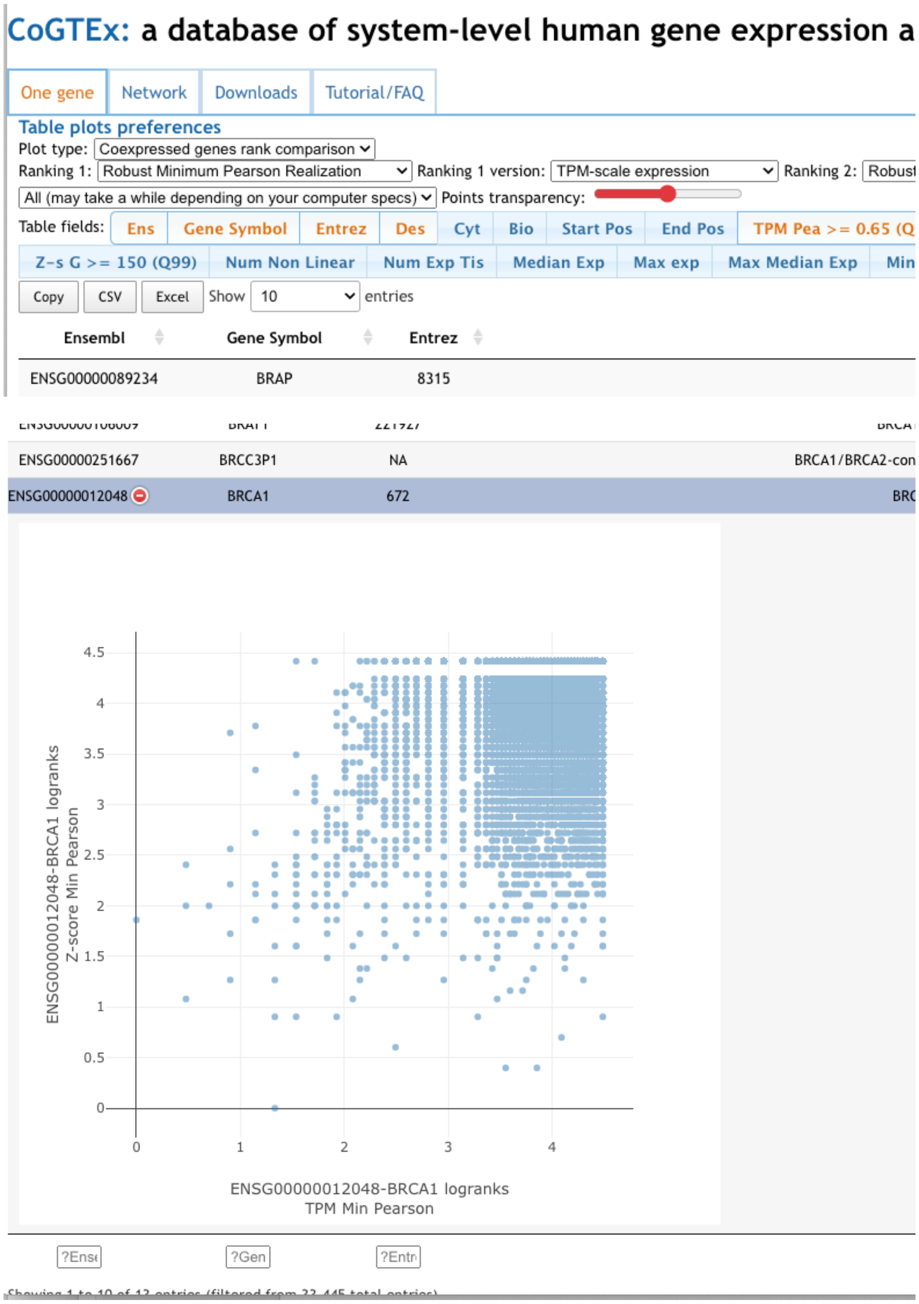

Supplement: Attachment — Submitted filename: Point-by-point answers CoGTEX PLOS-One-rev4.pdf [file pone.0309961.s004.pdf]
