# Supplementary material for: CoGTEx: Unscaled system-level coexpression estimation from GTEx data forecast novel functional gene partners
Source: PLoS One. 2024 Oct 4;19(10):e0309961. doi: 10.1371/journal.pone.0309961 (PMC11451983; doi:10.1371/journal.pone.0309961)
Supplement: Attachment — Submitted filename: PONE-D-23-33636 - Reponse To Reviewers Comments.docx [file pone.0309961.s002.docx]

This document shows the responses to comments from two reviewers for the manuscript “CoGTEx: Unscaled system-level coexpression estimation from GTEx data forecast novel functional gene partners” submitted and reviewed in PLOS One. The answers are written in green, the manuscript corrections in red and the original review/editorial comments in black. We would like to remind that we answered the comments from reviewer #1. However, the comments from **reviewer #2 DOES NOT CORRESPOND TO OUR MANUSCRIPT**, therefore we did not answer such comments but only marked as “not related to our manuscript”.

========================
ORIGINAL FEEDBACK MAIL
========================
PONE-D-23-33636
CoGTEx: Unscaled system-level coexpression estimation from GTEx data forecast novel functional gene partners

Reviewer #1: In this research, the authors performed coexpression analysis of GTEx data and built web-site that provides the analysis results interactively. For estimation of system level and tissue specific coexpression network, they applied PCA, KNN-clustering, and t-SNE methods, which identified 42 tissue clusters. For estimation of linear and non-linear coexpression relationships, Pearson CC, Spearman CC, and G-statistic were used. I think they reported interesting results, but I have some concerns to be addressed.

First, (a) it is difficult to understand the benchmark study of current research with GIANT and KEGG databases. When the enrichment analysis was applied with KEGG pathway information, I wonder whether (b) multiple testing correction was considered. (c) Moreover, they seemed to use the coexpression pairs in GIANT database to validate non-randomness of gene coepxressions that they found. I think this is not enough comparison to prove validity of their results, and another comparison should be performed. (d) For example, gene pair information about genetic regulatory network can be obtained from external databases such as GTRD, and this information should be ground-truth gene pairs. The comparisons with such gold standard examples would give more confidence about the current research.

**Authors Response:**

(a) We appreciate the comment of the difficulty. Therefore, we amended the corresponding section. First, we introduced the topic and refer other studies doing and recomending similar evaluations.

“Since one of the goals of coexpression analyses is to find possible functional partners. It is common to evaluate coexpressed genes with gene-pairs from known biological networks [32][28]”.

Second, we provide an example that clarifies the experiment. In the description of the results of figure 3ª regarding the KEGG information, we added:

“The results are shown in Figure 3A. For example, for *Pearson* TPM, the median is 7 associated KEGG pathways from the top 5% coexpressed genes. Moreover, 25% of the genes show more than 13 pathways associated. In comparison, 5% of bottom coexpressed genes, basically those showing no correlation, the median is only 3 pathways and marginal number of genes show more than 10 pathways. Therefore, overall, Figure 3A reveals that the distributions of the number of associations of top genes (red) are clearly more associated with KEGG canonical pathways than the bottom no coexpressed genes (blue) in the six comparisons.”.

Third, we clarify the caption of Figure 3. The current caption is:

“**Figure 3**. **Pathway and network enrichment analysis.** Panel A shows the number of associations (hypergeometric test p < 0.05) to KEGG pathways of the top 5% of most coexpressed genes per every gene compared to the bottom 5% (which should represent no correlation). Each boxplot is composed of the number of associations to ~200 KEGG pathways of the 33,445 genes considered. Panel B shows the AUROC per gene classifying positive and negative GIANT gene pairs from tissue-specific networks.”.

(b) We agree that multiple correction should be applied in a common functional analysis that is meant to be further described for a particular set of coexpressed genes. However, in this case, as specified in the text, we are using the p-value as an indicator of information content not as statistical test. We could also use, for instance, the ratio of the number of coexpressed pairs within a pathway (which would show basically the same result).

Indeed, using the 33,445 genes in our experiment is imprecise because there are many genes which their function is unknown and not represented in pathways, which would skew the distribution of associations. However, as said, the experiment is meant to show a tendency in information content instead of showing exact associations to pathways, so we did the estimation for all genes to avoid defining filters which would complicate even more the interpretation and the goal of the experiment.

(c) We are using GIANT, KEGG, literature for specific cases, and comparisons with other databases to support our estimations.

(d) We appreciate the reviewer’s comment regarding GTRD. GTRD is a very nice resource that link many types of experiments regarding gene-regulation, in particular for transcription factors and regulators.

To verify the utility of this information as a ground-truth, we queried in GTRD, 3 genetic regulators BRCA2, ARID4B, and FOXA1 for chromatin immuno-precipitation ChIP-Assays (we will refer to regulators instead of Transcription Factors -TF- because many experiments have been done on previously known as TF but some authors define them as not TF because of a DNA-site-specific criteria, see <http://humantfs.ccbr.utoronto.ca/index.php> from <https://pubmed.ncbi.nlm.nih.gov/29425488/>).

Then from the experiments linked in GTRD, we used those from GEO/NCBI which we found annotated information regarding the target genes to perform further comparisons. For BRCA2 we used GSE133450 (peaks in file GSE133450_BRCA2.anno.xlsx). For ARID4B, we used GSE148272, (peaks in file GSE148272_K562_ChIP-seq.xlsx), while for FOXA1 we used GSE101407 (the corresponding list of binding peaks in file GSE101407_annotated_1_4_6mergehomer_macs _peaks.xlsx). From these, there were 23317, 9860, and 11733 peaks for BRCA2, ARID4B, and FOXA1 respectively. These peaks correspond to 15632, 9860, and 6521 unique genes respectively (in the case of ARID4B, the data was already filtered by duplicated genes). Because the number of peaks in genes are huge, a hypergeometric test of top-coexpressed genes does not represent a full picture of the associations. Therefore, we drawn the ranks of co-expressed genes for Z-Score and TPM scales relative to annotations of peaks compared to the overall ratio of total genes overlapping in both databases (peaks and CoGTeX). As can be seen in the figure below, the overall peaks suggest that physical interaction is associated to coexpression but it is highly unspecific, and the magnitude depends on the regulator. That is, even that there is some tendency for coexpressed genes to show peaks, it is not definitive. The deviations from randomness (red-line) seems to be TF-specific but also biased by the cell-type used to estimate the ChIP assay. In conclusion, these data cannot be clearly used as ground-truth, mainly, because the number of peaks is huge and unspecific. Nevertheless, we added the figure in results because it shows a possible mechanism of coexpression. We also added a paragraph in discussion about this and a corresponding paragraph in methods.

Thus, the new section in results is:

Coexpression is associated to DNA-binding regulation

One of the possible mechanisms leading to coexpression is the regulation by TFs, cofactors, or regulators. To explore this possibility, we investigated three genes involved in genetic regulation where the peak results of the chromatin immuno-precipitation assays with sequencing (ChIP-Seq) and their corresponding annotated gene are available in the GEO database from NCBI. Specifically, we chose BRCA2, ARID4B, and FOXA1 and corresponding assays from GEO (see Methods). The number of unique genes showing a ChIP-Seq peak and included in our analyses correspond to 11488, 7846, and 5236 respectively. The expectation is that higher coexpresion should be more likely to show ChIP-Seq peaks. To show a full-picture of the peaks and coexpression, we compared the ranks of co-expressed genes with its annotation from the ChIP-Seq peaks. As can be seen in the Figure 7, there are clear deviations from randomness (represented by the red line), and there are denser peaks detected at the coexpressed genes (at the right) than peaks at the not coexpressed genes (at the left) for the three genes investigated and, at different degrees, in both z-score and TPM scales. The deviations from randomness seems to be regulator-specific although an additional bias should be attributed to the cell-type used to estimate the ChIP assay. Overall, these results support an association between coexpression and regulators bound at target genes, which seems stronger at coexpression from z-scores than from TPM scale, supporting the use of both estimations. Unfortunately, binding of regulators covers thousands of genes and appears highly unspecific relative to coexpression, thus binding seems to be only partially associated to coexpression.

**Figure 7**. **Comparison of coexpression and ChIP-Peaks for selected transcriptional regulators.** Each panel shows the ChIP-peaks for a regulator, in z-score at left, or TPM at right. Vertical black lines represent ChIP-peaks for the corresponding coexpressed gene. The height of each line corresponds to the cumulative number of genes showing a peak. The red line corresponds to the expected ratio, which is estimated by the total genes showing a peak divided by the 33,445 genes. Thus, for random order the height of black lines should match the red line as shown in the bottom panels for two random orders of genes (for ARID4B and FOXA1 data). Deviations from the red line should correspond to higher association between peaks and the order of co-expressed genes. To quantify the deviation, a “D” score was estimated by the sum of the absolute difference between the observed counts (cyan) and the expected counts (red) divided by the total area of the triangle. This D-score represent the fraction of the area that is deviated from the expected. The D-score of ARID4B in z-score is 0.3452 which is almost 100 times higher than random D=0.0035. The lowest D-score correspond to 0.0259 of BRCA2 in TPM scale, which is 5.5 times higher than random (mean of 100 random order peaks in BRCA2 is D=0.0047). Note that the three regulators used show deviations from randomness, although at different degrees.

In methods, we added:

**Analyses of chromatin immuno-precipitation assays with sequencing.** Data from GEO were used to extract peaks representing DNA binding for TF. The series files GSE133450 (peaks in file GSE133450_BRCA2.anno.xlsx), GSE148272, (peaks in file GSE148272_K562_ChIP-seq.xlsx), and GSE101407 (the corresponding list of binding peaks in file GSE101407_annotated_1_4_6mergehomer_macs _peaks.xlsx) were used for BRCA2, ARID4B, and FOXA1 respectively. From these data, there were 23317, 9860, and 11733 peaks for BRCA2, ARID4B, and FOXA1 respectively. These peaks correspond to 15632, 9860, and 6521 unique genes respectively (in the case of ARID4B, the data was already filtered by duplicated genes). Finally, from these annotated genes there were 11488, 7846, and 5236 genes respectively that were also included in our 33,445 genes analyzed for coexpression. For each TF, the CoGTeX list of all coexpressed genes were extracted from the web tool and sorted by absolute value. Then, the gene symbol was matched to the corresponding peak list and annotated whether the gene was present or not. Cumulative presence counts were then used and showed in figures.

And, finally, in Discussion we added:

We showed that transcriptional regulation is partially associated to system- and tissue-level coexpression. This result shows that some coexpressed genes are due to direct genetic regulation (e.g. TF🡪Gene). However, TF-binding or regulator bound to TFs seems highly unspecific, which could be due to the cell-type context or stimuli and the result of indirect or convergent genetic regulations. The indirect regulation occurring when a regulator appears coexpressed with a *Gene* through a second regulator (e.g. TF1🡪TF2🡪Gene) while the convergent resulting when *Gene1* is coexpressed with *Gene2* due to similar regulation (e.g. TF🡪Gene1 and TF🡪Gene2). In any case, system- and tissue-level coexpressions could provide clues or hypotheses of such regulations.

Second, comparison with COXPRESdb should be reconsidered in terms of above-mentioned issue. Moreover, they needs to compare the results with different thresholds, for example, with different thresholding of highly significant results, not only with top 100 results. The consistent results with different thresholds also improve the value of this research.

**Authors Response:**

As recommended, we re-run the analyses for different top number of genes. We extended the analysis of top 100 adding top 500 and top 1,000. These results are included in Supplementary Figure 6. The tendencies are conserved, thus the messages holds (that TPM ranks are different to z-score and that TPM coexpressions are obscured if only z-score is considered). We appreciate the comment that improved the information provided. We added

Third, since the different tissues were sampled in the same participant, there might be correlations between samples that were used in the determination of coexpressions between genes. Therefore, any methods breaking the correlations should be considered.

**Authors Response:**

Thanks for this comment that allow us to clarify our approach. In our estimations the possible marginal correlation within samples is broken in two ways. First, we describe in methods and in Figure 2 that for each calculation, there are 70 samples independently chosen randomly per tissue. Second, this procedure is performed 20 times and the lowest value from these 20 is used (as shown in Figure 2). Therefore, the possible marginal correlation from sample to sample across tissues is broken by these two factors.

Fourth, the authors presented the examples of different results between system level and tissue specific coexpressions. I think it would be better to describe possible mechanisms of such differences between tissues.

**Authors Response:**

The mechanisms explaining the differences are out of the scope of the current manuscript. The examples shown are meant to provide support that the system-level coexpression is different and can be biologically meaningful. This new type of coexpression is still an observation; we are not sure what are the mechanisms behind it. Indeed, one of our goals is to let the scientific community use the tool and explore and provide mechanistic insights in particular gene-modules, which will help to broad our knowledge of regulation at the system-level.

Therefore, we added the following paragraph in the discussion:

What are the mechanisms leading to system-level coexpression? As in tissue-level coexpression, the expectative is that there is a genetic control behind. We have shown examples where the mean level, and therefore system-level coexpression, is important (Figure 5 and Supplementary Material). By now, our database will be useful to provide insights and hypotheses, which may lead to help uncover novel biological mechanisms.

**NOTE:** The comments from **reviewer #2 DOES NOT CORRESPOND TO OUR MANUSCRIPT**, therefore we did not answer such comments but only marked as “not related to our manuscript”.

**Reviewer #2**: In this study, the authors used transcriptomics and eFC approaches to investigate
the biological mechanisms of depression. They found altered eFC patterns in specific
brain regions of patients with MDD and investigated their association with gene expression changes in the brain.

**This comment from reviewer #2 IS NOT RELATED TO OUR MANUSCRIPT regarding COGTEX.**

I am glad I had the opportunity to review this paper.
Despite the effort of the authors to be rigorous and objective, especially in the neuroimaging section, I believe there are flaws in the biological characterization of the genes involved in the identified eFC alterations that prevent me from supporting the idea that this paper “bridged the gap between transcriptomic and neuroimaging” or contributed to what the authors state as “a deep understanding of the development of disease from a molecular perspective”.

**This comment from reviewer #2 IS NOT RELATED TO OUR MANUSCRIPT regarding COGTEX.**

MAJOR


1) The authors describe the construction of the eFC starting by dividing the brain into 90 regions, and then, to estimate the case-control eFC differences, they perform T-tests on standard entropy values for each brain region. Afterward, they go on to examine the association between the healthy brain transcriptome and regional changes in the eFC of MDD by using partial least squares regression (PLS) and the 45 regions × 15633 gene expression levels matrix from the AHBA.
When they report in the Results section (page 14, line 265) that 618 genes constituted the regional change in the eFC gene list in individuals with MDD, it was not clear whether these genes were associated with the eFC of the regions in common between the input dataset and the AHBA or instead were associated with only the regions whose eFC was significantly differentially altered between HC and MDD. If the former is the case, then it would be difficult to ascribe the role of these genes only to the MDD-specific eFC alterations.

**Authors Response: This comment from reviewer #2 is not related to our CoGTEX manuscript.**

2) The authors focus their transcriptomic analysis on the gene expression values of the first component of PLS (PLS1) that showed the strongest correlation with regional changes in eFC. Then, they select genes with large positive PLS1 weights that correspond to genes with higher-than-average expression levels in regions where eFC increases and genes with lower-than-average expression levels in regions where eFC decreases.
Why did the authors not consider the reverse relationship between gene expression and eFC patterns? It is plausible to find eFC increases/decreases caused by lower/higher gene expression, respectively.

**Authors Response: This comment from reviewer #2 is not related to our CoGTEX manuscript.**

3) In the Results section, the authors report 13 MDD-related genes associated with changes in eFC obtained by overlapping the genes identified in PLS analysis with MDD-related genes (Zeng et al. 2012).
How did you define the statistical significance of this overlap?
Why did you not analyze the 269 genes identified in the latest GWAS of MDD (Howard et al. 2019) as a more accurate list of MDD risk-related genes?
Did you consider testing other pathologies risk-related genes (SCZ, BD, OCD, ASD) to rule out the plausible pleiotropy of the genes identified?
Indeed, most of the brain regions in which the eFC alterations were found are overlapping between these disorders (especially with SCZ).
Moreover, in the same paragraph, authors do not specify the statistical test implemented to examine the “relationships between overlapped gene expression and case-control changes in eFC”.

**Authors Response: This comment from reviewer #2 is not related to our CoGTEX manuscript.**

4) Further, many GO terms identified are basically related to most common biological processes found in every brain cell-type (regulation of dephosphorylation, regulation of cellular localization and cell cycle phase transition, chemical synaptic transmission, dephosphorylation, regulation of phospholipid biosynthetic process) with no specific evidence of MDD-related processes.
Additionally, no statistical test was implemented to evaluate the replication of the 13 MDD-related genes in the external datasets nor of the 31 hub genes identified in the PPI network analysis.
There is no substantial evidence that MAPK and cAMP signal pathway disruptions are related to the underlying molecular mechanisms of MDD instead of another complex psychiatric disorder. Thus, I found it very difficult to support the following statement on page 21, line 420: “These findings suggest the central role of these hub genes in the genetic mechanisms of depression”.

**Authors Response: This comment from reviewer #2 is not related to our CoGTEX manuscript.**

5) Finally, ACC, a region critically involved in mood disorders and whose FC alterations have been found in patients with MDD (de Kwaasteniet et al. 2013; Qiu et al. 2020; Hu et al. 2022), was not found among the regional eFC changes.
It would be worth elaborating in the discussion on the possible reason behind this unexpected lack of evidence.

**Authors Response: This comment from reviewer #2 is not related to our CoGTEX manuscript.**

6) Overall, the paper should be more focused on what can be achieved with this new eFC approach and its potential to help understand the biological mechanisms underlying MDD in light of the limitations of the study, and I would urge the authors to be very cautious about the take-home message delivered, as I reckon a clear and reliable connection to the pathology underlying mechanisms is still missing.

**Authors Response: This comment from reviewer #2 is not related to our CoGTEX manuscript.**

MINOR
7) The abstract lacks clarification of the specific findings that led the authors to conclude that they provide new insights into the underlying neurobiological mechanisms of MDD. On page 4, lines 46-48, they do not specify where the genes they mention come from nor their biological function, and they merely report that they “investigated the biological pathways with enrichment analysis” without describing the results they obtained. The conclusion section should be rewritten in a less superficial manner considering the results obtained.

**Authors Response: This comment from reviewer #2 is not related to our CoGTEX manuscript.**

8) Page 4, line 30. I would use the term alterations instead of aberrancies when referring to brain networks.

**Authors Response: This comment from reviewer #2 is not related to our CoGTEX manuscript.**

9) It is not clear what the authors mean with the term “global” on page 5, line 57 when referred to MDD. Is it referred to the global aspects of everyday life that are affected, or to the global brain functions that are impaired in patients with MDD (mainly emotion and cognitive functions) or to a more general epidemiological aspect? I would suggest clarifying this point.

**Authors Response: This comment from reviewer #2 is not related to our CoGTEX manuscript.**

10) Details about the type of gene expression measurements used (RPKM, FPKM, TPM), number of genes and samples filtered is missing in Supplementary materials.

**Authors Response: This comment from reviewer #2 is not related to our CoGTEX manuscript.**

11) The cell-type enrichment analysis needs better explanation.
It is not clear to me how the average gene expression for each cell-class gene set in each of the AHBA 45 regions was used in the enrichment analysis. What overrepresentation test was used (hypergeometric, permutation, Fisher exact test)?
Which universe was used as null comparison?

**Authors Response: This comment from reviewer #2 is not related to our CoGTEX manuscript.**

12) The authors reported the use of k-means clustering to define brain regions communities based on the eFC matrix and decided to study only k=10 communities based on Faskowitz et al. 2020 results.
Nevertheless, It is not mentioned by the authors whether they applied the same exact two-step procedure reported by Faskowitz et al., which consists in performing k-means clustering on the top 50 PCs of the eFC matrix, or they opted for a different strategy.
Moreover, I understand the decision to use this algorithm over others as it exhibits significantly faster runtimes, but perhaps it is worth evaluating at least the optimal number of clusters that better explains the variance in your data (elbow method, gap-statistic, silhouette method), instead of picking the k=10 evaluated in a different dataset and study.

**Authors Response: This comment from reviewer #2 is not related to our CoGTEX manuscript.**

- - - - -
For journal use only: PONEDEC3
